# Can Intrapartum Cardiotocography Predict Uterine Rupture among Women with Prior Caesarean Delivery?: A Population Based Case-Control Study

**DOI:** 10.1371/journal.pone.0146347

**Published:** 2016-02-12

**Authors:** Malene M. Andersen, Dorthe L. A. Thisted, Isis Amer-Wåhlin, Lone Krebs, The Danish CTG Monitoring during VBAC study group

**Affiliations:** 1 Dept. of Obstetrics and Gynaecology, University of Copenhagen, Holbaek Hospital, Holbaek, Denmark; 2 University of Copenhagen, Hvidovre Hospital, Dept. of Obstetric and Gynecology, Hvidovre, Denmark; 3 Dept. of Women and Child Health, Karolinska Institute, Stockholm, Sweden; Indiana University School of Medicine, UNITED STATES

## Abstract

**Objective:**

To compare cardiotocographic abnormalities recorded during labour in women with prior caesarean delivery (CD) and complete uterine rupture with those recorded in controls with prior CD without uterine rupture.

**Study Design:**

Women with complete uterine rupture during labour between 1997 and 2008 were identified in the Danish Medical Birth Registry (*n* = 181). Cases were validated by review of medical records and 53 cases with prior CD, trial of labour, available cardiotocogram (CTG) and complete uterine rupture were included and compared with 43 controls with prior CD, trial of labour and available CTG. The CTG tracings were assessed by 19 independent experts divided into groups of three different experts for each tracing. The assessors were blinded to group, outcome and clinical data. They analyzed occurrence of defined abnormalities and classified the traces as normal, suspicious, pathological or pre-terminal according to international guidelines (FIGO).

**Results:**

A pathological CTG during the first stage of labour was present in 77% of cases and in 53% of the controls (OR 2.58 [CI: 0.96–6.94] *P* = 0.066). Fetal tachycardia was more frequent in cases with uterine rupture (OR 2.50 [CI: 1.0–6.26] *P* = 0.053). Significantly more cases showed more than 10 severe variable decelerations compared with controls (OR 22 [CI: 1.54–314.2] *P* = 0.022). Uterine tachysystole was not correlated with the presence of uterine rupture.

**Conclusion:**

A pathological cardiotocogram should lead to particular attention on threatening uterine rupture but cannot be considered a strong predictor as it is common in all women with trial of labour after caesarean delivery.

## Introduction

Complete uterine rupture is a rare but severe obstetric complication which, in Denmark, affects approximately 0.45% of women with intended vaginal birth after a caesarean (VBAC)[1]. Since the increased risk of uterine rupture is small and a planned vaginal delivery presents benefits in term of reduced maternal morbidity, a prior caesarean delivery (CD) is generally not regarded as a contraindication to vaginal birth in Denmark[2] or in Scandinavia in general.

Fetal heart rate changes in terms of severe bradycardia or undetectable fetal heart rate and the cessation of contractions and/or loss of station of the presenting part of the fetus are well-known symptoms of complete uterine rupture[3,4]. Even if an emergency CD is performed, the risk of adverse perinatal outcome and severe maternal morbidity is very high[3,5,6]. Intervention before the catastrophe occurs is of crucial importance, and thus identifying the early signs of uterine rupture during labour is essential.

Signs of threatening uterine rupture in terms of abdominal pain, vaginal bleeding, and various cardiotocographic abnormalities are difficult to interpret and evaluate because they are very common[3,7].

Most studies of cardiotocographic abnormalities have evaluated the sensitivity and the specificity of the method in predicting fetal acidemia. Overall intrapartum monitoring with cardiotocography has a high sensitivity but a low specificity because abnormalities are very common[8]. The specificity can be increased by combined use with automatic ST waveform analyses (STAN)[9].

Only a few studies have evaluated the use of cardiotocography during a trial of labour after caesarean (TOLAC), and the results are conflicting[10,11]. In Denmark, national guidelines recommend continuous fetal heart tracing during a trial of labour after a CD[2]. These tracings are interpreted according to international guidelines,[12] but specific guidelines regarding the interpretation of fetal heart tracings during a VBAC do not exist. However, recommendations in guidelines for TOLAC imply that changes in the fetal heart tracings are the most frequent and sometimes the only indicator of a rupturing uterus[2,4].

The objective of this study was to compare cardiotocogram (CTG) tracings in women undergoing TOLAC with and without uterine rupture, with the aim to identify changes that could have predicted the forthcoming uterine rupture.

## Materials and Methods

Cases with prior CD and uterine rupture during labour at term reported to the Danish Medical Birth Registry between 1997 and 2008 were reviewed. All cases with complete uterine rupture and an available CTG were included. A complete uterine rupture was defined as a full separation of the uterine wall (including the overlying serosa) and involvement of fetal membranes, resulting in a direct communication between the uterine cavity and peritoneum.

During the study period, 62,475 women with a singleton pregnancy and a history of CD were identified. A CD was planned in 19,767 (32%) of these women, and a TOLAC in the remaining 39,042 (63%) women. A complete uterine rupture at term occurred in 181 (0.46%) cases. A control group was selected by identifying the subsequent two intended VBAC´s in the Medical Birth Registry for every case. CTG tracing were available in 60 cases and 55 controls.

**Fig 1:**
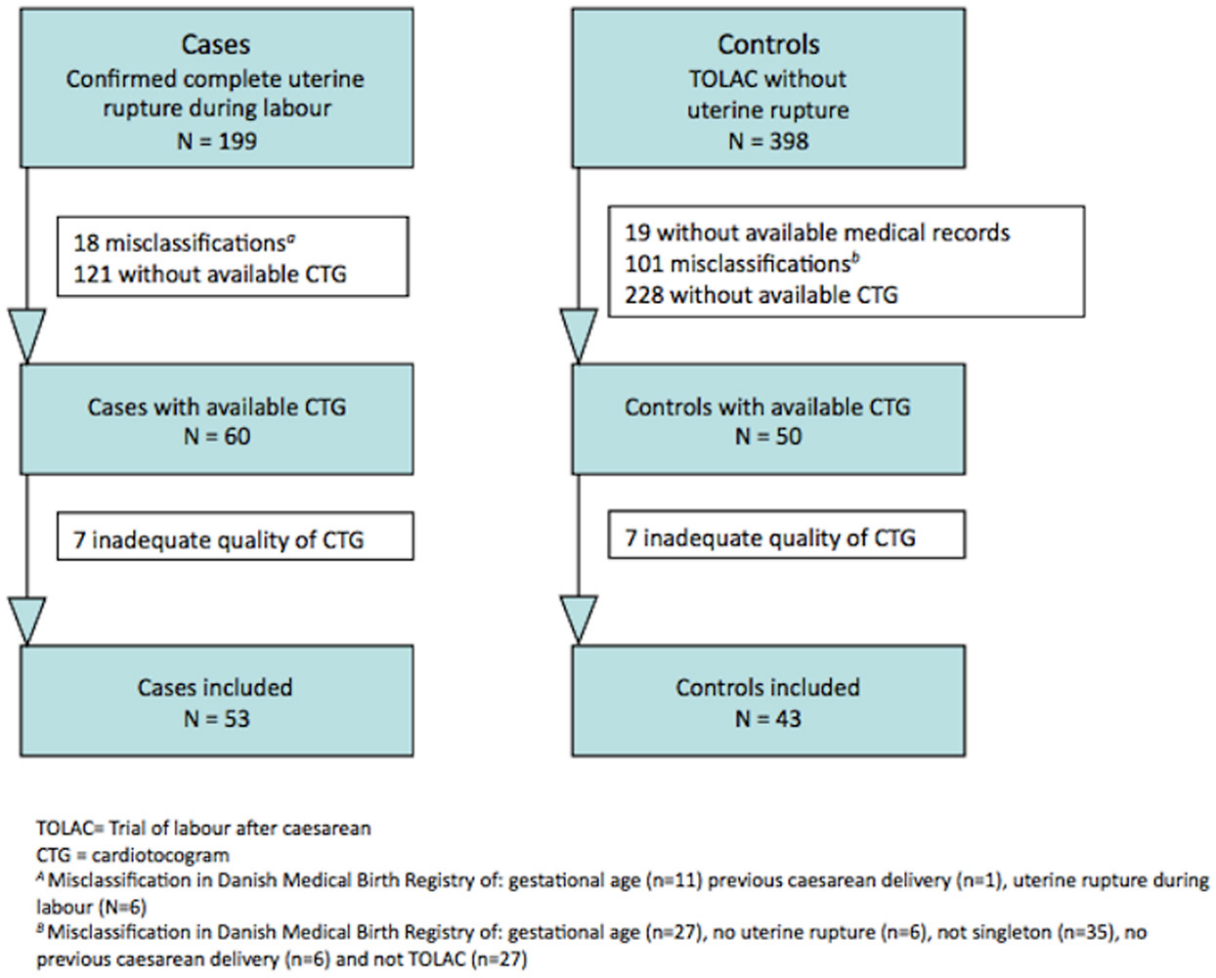
Flow diagram of cases and controls included in the study.

The included cases and controls with available CTG tracings delivered in 16 different departments. A total of 53 cases and 43 controls met the criteria (Fig 1).

CTG tracings from both cases and controls were anonymised and randomly assigned with a study number. CTGs that were technical unreadable according to the authors objective judgement were excluded. Only tracings from the last 4 hours prior to delivery were included. If the CTG tracing ended with terminal bradycardia, this part of the tracing was blinded or removed with the aim of completely blinding the assessor to outcome.

An evaluation scheme was developed based on the International Federation of Gynaecology and Obstetrics (FIGO) guidelines (12) (S1 Appendix and S2 Appendix), and each CTG was prepared identically in three copies, with one evaluation scheme enclosed per CTG page. Each page contained CTG tracing of 30 minutes. The assessors were asked to evaluate the CTG and classify it as normal, suspicious, pathological or pre-terminal. Furthermore, a description of the occurrence of different abnormalities was requested, including an evaluation of signs of tachysystole, defined as more than five contractions in 10 minutes. In cases in which STAN surveillance had been used, the ST analysis was not assessable for evaluation.

We recruited 19 blinded and independent experienced obstetricians from leading obstetric departments in Denmark and Sweden (The Danish CTG Monitoring during VBAC Study Group). They were all subspecialized within obstetrics and had more than ten years of clinical experience. They were asked to examine approximately 20 randomly selected CTGs. Assessors from the same obstetrical department did not evaluate the same CTGs. Three different assessors evaluated each CTG tracing.

When the evaluation schemes were returned, all data was entered in SPSS 20.0 Statistics (IBM). Two of the authors (Malene Andersen and Dorthe Thisted) independently entered all data in the database. Subsequently data was merged and in case of disagreement corrected by use of information from the evaluation schemes. Data yielded variables that allowed a quantitative list of a specific abnormality. For each tracing, mean values for each variable were calculated based on the three different evaluations.

Statistic analyses were performed using 2 x 2 tables to calculate odds ratios. An independent sample *t*-test was used when comparing mean values. Tests were considered statistically significant when *P* < .05.

The study was approved by the Danish Data Protection Agency (Journal number: J.nr. 2008-41-2256) and the National Board of Health approved access to medical records (J.nr. 3-3013-168/1).

## Results

There were no significant differences between cases and controls with regards to maternal demographic characteristics. In particular, there were no differences in the number of previous caesarean or vaginal deliveries (Table 1).

**Table 1:** Baseline characteristics of cases and controls.

	Cases (n = 53)	Controls (n = 43)	*P*
**Prelabour characteristics:**			
Maternal age^1^	32.5 (28–44)	33.34 (23–43)	0.334
Parity^1^	1.53 (1–5)	1.64 (1–4)	0.502
Previous CD^1^^,^^2^	1.00 (1)	1.02 (1–2)	0.269
No previous vaginal deliveries^3^	46 (87%)	33 (77%)	0.216
BMI^1^^,^^4^	24.81 (18.15–37.11)	24.84 (19.26–37.2)	0.980
Gestational age^1^	40+4 (37+1–42+4)	40+4 (37+5–42+2)	0.725

^1^Data are presented as mean (range).

^2^ CD: caesarean delivery

^3^Data are presented as number (percentages).

^4^ BMI: body mass index

During labour epidural anaesthesia and augmentation by oxytocin were used more frequently in cases than in controls. There was no difference in rate of induction or number of fetal scalp pH samples during labour (Table 2).

**Table 2:** Baseline characteristics of labour in cases and controls.

	Cases (n = 53)	Controls (n = 43)	OR	95% CI	*P*
Labour characteristics^1^:					
Induction of labour	19 (36%)	14 (33%)	1.16	0.49–2.71	0.743
Epidural	31 (58%)	17 (40%)	2.16	0.95–4.89	0.070
Augmentation by oxytocin	36 (68%)	22 (51%)	2.01	0.88–4.64	0.102
Augmentation 0–2 hours	12 (23%)	13 (30%)	1.14	0.41–3.14	0.805
Augmentation > 2 hours	24 (45%)	9 (21%)	3.29	1.21–8.94	0.020
Fetal scalp pH	11 (21%)	10 (23%)	0.86	0.33–2.28	0.772
Fetal scalp pH ≥3	3 (6%)	2 (5%)	1.18	0.19–7.47	0.887
Duration of labour:					
Duration of stage 1 (min.)^2^	355 (23–1293)	254 (25–845)			
Stage 1 ≥ 8 hours^1^	12 (23%)	3 (7%)	4.43	1.13–17.33	0.027
Duration of stage 2 (min.)^2^	64 (10–198)	71 (5–245)			
Stage 2 ≥ 1 hour^1^	5 (28%)	11 (32%)	0.74	0.20–2.72	0.676
Stage 2 ≥ 2 hours^1^	2 (11%)	5 (15%)	0.65	0.11–3.97	0.688
Duration of labour, stages 1 & 2 (min.)^2^	377 (23–1293)	310 (50–928)			
Delivery:					
Dilation of cervix (cm.)^2^	7.0 (1–10)	9.58 (1–10)			
Fully dilated^1^	19 (36%)	34 (79%)	0.15	0.06–0.37	< .001
Caesarean section in 1st stage^1^	35 (66%)	9 (21%)	7.35	2.90–18.6	< .001
Caesarean section in 2nd stage^1^	14 (78%)	2 (6%)	56	9.17–342.1	< .001

^1^Data are presented as number (percentages).

^2^Data are presented as mean (range).

Only four (8%) of the 53 cases delivered vaginally compared with 32 (74%) of the controls. CD was performed in 35 (66%) of the cases during the first stage of labour. Of the 18 women that reached the second stage 14 (78%) delivered by CD Among the controls, 11 (26%) delivered by caesarean, the majority (82%) during the first stage of labour.

There was a trend towards cases having a longer duration of the first stage of labour (Table 2). There was no difference between duration of the second stage of labour between cases and controls.

Surveillance with STAN with addition of ST analysis to CTG was used in eight (15%) cases and seven (16%) controls.

**Fig 2:**
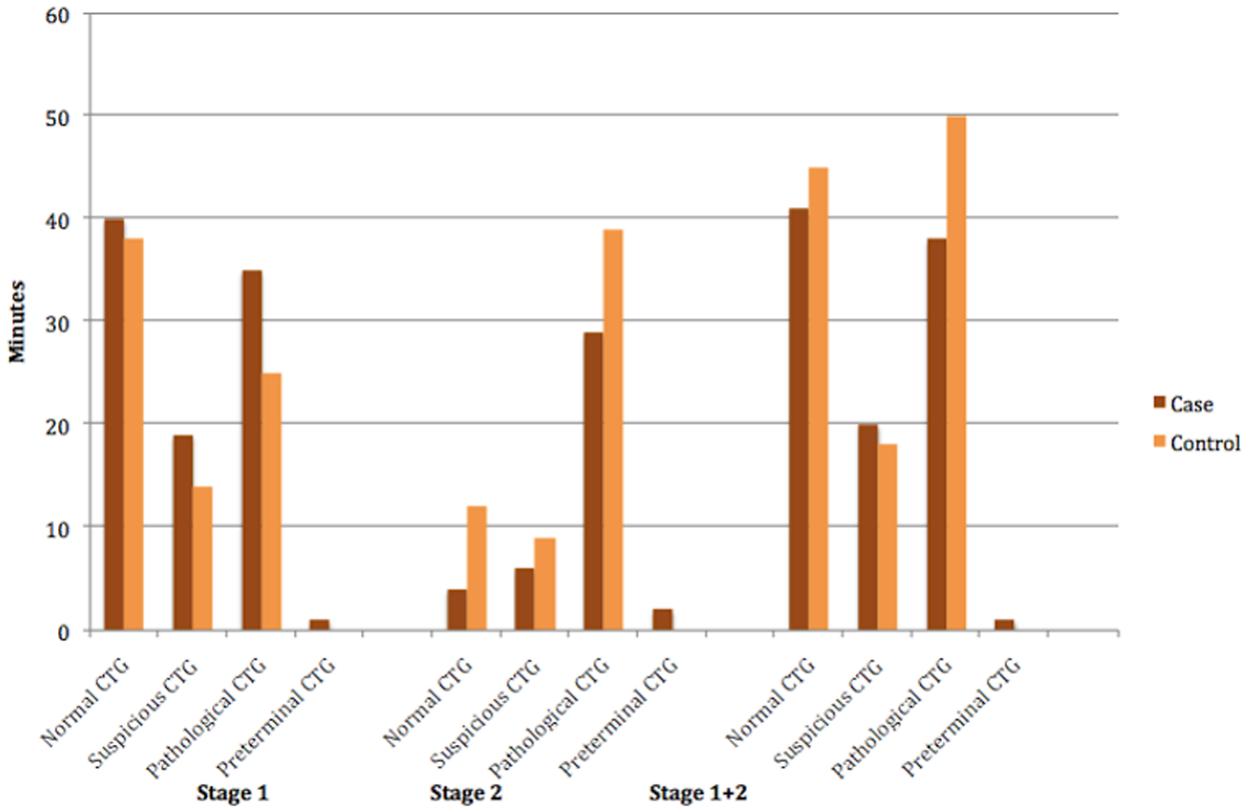
Durations of cardiotocographic (CTG) changes.

CTG changes were frequent in cases as well as in controls in the last 4 hours of labour (Tables 3 and 4 and Fig 2). Only two of the controls (5%) and none of the cases had a completely normal trace during labour. During the first stage of labour, suspicious CTG was present in approximately half of the tracings from both cases and controls (Table 3). Likewise, a pathological CTG was present in 41 (77%) of the cases and 23 (53%) of the controls. Preterminal tracings existed only among cases.

**Table 3:** Cardiotocographic (CTG) changes in cases and controls during labour.

Stage 1	Cases (*n* = 53)	Controls (*n* = 43)	OR	95% CI	*P*
**Classification of CTG**^A^					
No presence of normal CTG (ref.)	13 (25%)	8 (19%)	Ref		
Presence of normal CTG	36 (68%)	28 (65%)	0.79	0.29–2.17	0.664
No presence of suspicious CTG (ref.)	22 (42%)	15 (35%)	Ref		
Presence of suspicious CTG	28 (53%)	21 (49%)	0.91	0.38–2.16	0.835
No presence of pathological CTG (ref.)	9 (17%)	13 (30%)	Ref		
Presence of pathological CTG	41 (77%)	23 (53%)	2.58	0.96–6.94	0.066
Pathological CTG < 60 min.	31 (76%)	18 (78%)	2.49	0.89–6.96	0.089
Pathological CTG ≥ 60	10 (24%)	5 (22%)	2.89	0.73–11.36	0.144
No presence of preterminal CTG (ref.)	46 (87%)	36 (84%)	Ref		
Presence of preterminal CTG	3 (6%)	0	NA		
**Tachycardia**^A^					
No presence of tachycardia (ref.)	25 (47%)	26 (60%)	Ref		
Presence of tachycardia	24 (45%)	10 (23%)	2.50	1.00–6.26	0.053
Tachycardia < 60 min.	15 (63%)	6 (60%)	2.60	0.87–7.77	0.090
Tachycardia ≥ 60 min	9 (38%)	4 (40%)	2.34	0.63–8.58	0.212
**Variability**^A^					
No periods of impaired variability (ref.)	27 (51%)	21 (49%)	Ref		
Presence of impaired variability	23 (43%)	15 (35%)	1.19	0.50–2.83	0.698
Impaired variability < 40 min	16 (30%)	14 (33%)	0.89	0.35–2.22	0.805
Impaired variability ≥ 40 min	7 (13%)	1 (2%)	5.44	0.62–47.75	0.109
**Decelerations**^A^					
No decelerations–any kind (ref.)	1 (2%)	4 (9%)	Ref		
Decelerations–any kind	49 (92%)	32 (74%)	6.13	0.65–57.3	0.106
Late decelerations	7 (13%)	5 (12%)	5.6	0.47–66.44	0.204
Severe variable decelerations	37 (70%)	22 (51%)	6.73	0.71–64.07	0.092
< 10 severe variable decelerations	26 (49%)	20 (47%)	5.2	0.54–50.2	0.158
≥ 10 severe variable decelerations	11 (21%)	2 (5%)	22	1.54–314.2	0.022

^A^ Data are presented as number (percentages).

**Table 4:** Cardiotocographic (CTG) changes in cases and controls during labour.

Stage 2	Cases (*n* = 18)	Controls(*n* = 34)	OR	95% CI	*P*
Drop-outs	4^1^	3^2^			
**Classification of CTG**^A^					
No presence of normal CTG (ref.)	10 (56%)	18 (53%)	Ref		
Presence of normal CTG	3 (17%)	13 (38%)	0.42	0.10–1.81	0.261
No presence of suspicious CTG (ref.)	9 (50%)	18 (53%)	Ref		
Presence of suspicious CTG	4 (22%)	13 (38%)	0.62	0.16–2.44	0.516
No presence of pathological CTG (ref.)	1 (6%)	3 (9%)	Ref		
Presence of pathological CTG	13 (72%)	28 (82%)	1.39	0.13–14.7	0.845
No presence of preterminal CTG (ref.)	11 (61%)	31 (91%)	Ref		
Presence of preterminal CTG	2 (11%)	0	NA		
**Tachycardia**^A^					
No presence of tachycardia (ref.)	7 (39%)	16 (47%)	Ref		
Presence of tachycardia	7 (39%)	15 (44%)	1.07	0.30–3.77	0.924
Tachycardia < 60 min.	6 (86%)	13 (87%)	1.06	0.28–3.92	0.937
Tachycardia ≥ 60 min	1 (14%)	2 (13%)	1.14	0.09–14.77	0.902
**Variability**^A^					
No presence of impaired variability (ref.)	7 (39%)	16 (47%)	Ref		
Presence of impaired variability	7 (39%)	14 (41%)	1.14	0.32–4.07	0.844
Impaired variability < 40 min	6 (33%)	12 (35%)	1.14	0.30–4.29	0.849
Impaired variability ≥ 40 min	1 (6%)	2 (6%)	1.14	0.09–14.77	0.902
**Decelerations**^A^					
No decelerations–any kind (ref.)	1 (6%)	1 (3%)	Ref		
Decelerations–any kind	13 (72%)	30 (88%)	0.43	0.03–7.47	0.622
Late decelerations	3 (17%)	0	NA		
Severe variable decelerations	11 (61%)	25 (74%)	2.16	0.26–56.83	0.552
< 10 severe variable decelerations	8 (44%)	16 (47%)	0.5	0.03–9.08	0.692
≥ 10 severe variable decelerations	3 (17%)	9 (26%)	0.33	0.02–7.14	0.571

^A^ Data are presented as number (percentages).

^1^CTG tracing blinded for interpretation due to terminal bradycardia.

^2^CTG tracings not suitable for assessment because of poor quality.

There was a trend towards tachycardia occurring more frequently in CTG tracings from cases with uterine rupture (OR 2.50 [CI: 1.0–6.26] *P* = 0.053) in the first stage of labour. Also impaired variability for 40 minutes or more, was more frequently present in cases. Signs of hypertonia and increased variability were not correlated with uterine rupture.

In the first stage of labour, decelerations were present in 49 (92%) of the CTG tracings from cases and in 32 (74%) from controls. Severe variable decelerations were more frequently present in cases, 37 (70%) vs. 22 (51%), and there was a significant difference in the presence of 10 or more severe variable decelerations (OR 22 [CI: 1.54–314.2] *P* = 0.022).

There were no significant differences regarding the presence of CTG changes during the second stage of labour (Table 4).

Terminal bradycardia was present and blinded in 25 (47%) tracings from cases and in none in the control group. The average duration of terminal bradycardia in cases was 7 minutes.

Uterine tachysystole was common in both groups (Table 5). In the first stage of labour 51% of the cases versus 40% of the controls demonstrated presence of more than five contractions per ten minutes. In the second stage, uterine tachysystole was present in 50% of the cases and 53% of the controls (Table 5).

**Table 5:** Uterine tachysystole defined as more than five contractions per 10 minutes among cases and controls during labour.

Tachysystole	Cases (*n* = 53)		Controls (*n* = 43)	OR	95% CI	*P*
**Total**^A^	**1st stage**	(*n* = 53)	(*n* = 43)			
No (ref.)	22 (42%)		16 (37%)	NA		
Yes	27 (51%)		17 (40%)	1.16	0.48–2.80	0.755
**Total**^A^	**2nd stage**	(*n* = 18)	(*n* = 34)			
No (ref.)	3 (17%)		12 (35%)	NA		
Yes	9 (50%)		18 (53%)	2	0.45–8.94	0.392
**Total**^A^	**1st and 2nd stage**	(*n* = 53)	(*n* = 43)			
No (ref.)	21 (40%)		18 (42%)	NA		
Yes	31 (58%)		23 (53%)	1.16	0.50–2.65	0.738
**Induced**^A^	**1st stage**	(*n* = 19)	(*n* = 14)			
No (ref.)	6 (32%)		4 (29%)	NA		
Yes	12 (63%)		6 (43%)	1.33	0.27–6.61	0.740
**Induced**^A^	**2nd stage**	(*n* = 4)	(*n* = 12)			
No (Ref.	1 (25%)		5 (42%)	NA		
Yes	3 (75%)		7 (58%)	2.14	0.17–27.1	0.626
**Induced**^A^	**1st and 2nd stage**	(*n* = 19)	(*n* = 14)			
No (ref.)	6 (32%)		5 (36%)	NA		
Yes	13 (68%)		8 (57%)	1.35	0.31–5.94	0.704
**Stimulated (not induced)**^A^	**1st stage**	(*n* = 22)	(*n* = 8)			
No (ref.)	9 (41%)		4 (50%)	NA		
Yes	12 (55%)		4 (50%)	1.33	0.26–6.83	0.748
**Stimulated (not induced)**^A^	**2nd stage**	(*n* = 8)	(*n* = 9)			
No (ref.)	1 (13%)		2 (22%)	NA		
Yes	5 (63%)		7 (78%)	1.43	0.10–20.43	0.844
**Stimulated (not induced)**^A^	**1st and 2nd stage**	(*n* = 24)	(*n* = 12)			
No (ref.)	8 (33%)		5 (42%)	NA		
Yes	15 (63%)		7 (58%)	1.34	0.32–5.61	0.704
**Uterine tachysystole in the hour prior to rupture**^1^						
**Cases**^A^	Total		Inducted	Stimulated (not inducted)		
No	26 (49%)		11 (42%)	10 (38%)		
Yes	12 (23%)		4 (33%)	7 (58%)		
Missing data^2^	15 (28%)		4 (27%)	7 (47%)		

^A^Data are presented as number (percentages).

^1^Defined as one hour prior to the decision of performing an acute CD or time of birth.

^2^Due to non-assessable CTG tracings.

In the first stage of labour, uterine tachysystole was related to augmentation by oxytocin in 12 cases (55%) compared to four controls (50%). Among cases, 12 (63%) of the women with uterine tachysystole had had their labour induced. Among cases without uterine tachysystole, only six (32%) had had their labour induced (Table 5).

In the last hour prior to uterine rupture, uterine tachysystole was present in 12 cases (23%) and absent in 26 (49%). In 15 (28%) of cases the uterine contractions could not be evaluated due to poor quality of the CTG.

Perinatal morbidity and mortality was high when labour was complicated by complete uterine rupture (Table 6).

**Table 6:** Short-term neonatal outcome.

	Cases (*n* = 53)	Controls (*n* = 43)	*P*
Short-term outcome:			
Newborn weight (gram)^1^	3771 (2550–4945)	3533 (2590–4800)	< 0.001
Apgar score (5 min.) > 7^2^	32 (60%)	40 (93%)	NA
Apgar score (5 min.) ≤ 7^2^	20 (38%)	2 (5%)	< 0.001
Missing cases^2^	1 (2%)	1 (2%)	NA
Umbilical cord pH^1^	7.06 (6.57–7.39)	7.24 (6.88–7.36)	< 0.001
pH > 7.00^2^	27 (51%)	26 (61%)	NA
pH ≤ 7.00^2^	18 (34%)	1 (2%)	< 0.001
Missing cases^2^	8 (15%)	16 (37%)	NA
Perinatal deaths:			
Stillborn^2^	1 (2%)	1 (2%)	NA
Death within first week^2^	4 (8%)^3^	0	NA

^1^Data are presented as mean (range).

^2^Data are presented as number (percentages).

^3^Cause of death was complications to birth asphyxia.

## Discussion

This case-control study demonstrated that CTG abnormalities were very common in CTG tracings from women undergoing TOLAC. A pathological CTG was observed in more than half of the tracings, both in cases with a complete uterine rupture (77% in the first stage and 72% in the second stage of labour) and in controls without a rupture (53% in the first stage and 82% in the second stage of labour). A great number of severe variable decelerations in the first stage of labour may be a sign of threatening uterine rupture, but it was also a common finding in CTG tracings from women with planned VBAC without uterine rupture. Increased uterine activity did not seem to be a predictor of complete uterus rupture.

Most other studies have evaluated signs of true uterine rupture and found that severe bradycardia is strongly correlated[10,11,13]. In accordance with this, in none of our controls and in 25 (47%) of our cases did the tracings end with fetal bradycardia. A general recommendation exists of continuous surveillance with CTG during TOLAC[2,4]. Nevertheless, incorrect interpretation and/or an inappropriate response to CTG changes has been reported in large clinical series,[14] and it has been difficult to demonstrate that intrapartum CTG has any major impact on important obstetric indicators, with the exception of increasing operative delivery rates[15].

The present study was set up to evaluate the usefulness of CTG changes to diagnose a threatening uterine rupture in order to allow intervention before the complete uterine rupture occurs. A correct identification of the degree of clinical urgency could shorten the decision-to-delivery interval in emergency CD, and maybe even avoid general anaesthesia, and thus limit the risk of the poor perinatal outcome associated with complete uterine rupture.

Ridgeway et al. [10] studied fetal heart rate (FHR) patterns in 36 women with complete uterine rupture and 100 controls with a successful VBAC. They found no significant differences in fetal tachycardia, mild-moderate or severe decelerations, late or prolonged decelerations, or loss of uterine tone. Only presence of fetal bradycardia was significantly different between women with uterine rupture and women with a successful VBAC without uterine rupture.

In a review by Ayres et al., [11] 88% of women experiencing uterine rupture had recurrent late decelerations. The study was performed on tracings from only eight women with a complete uterine rupture, and women with none or up to six previous CD. No control group was used.

We found a trend towards both tachycardia and impaired variability for more than 40 minutes, were more often present in cases with a complete uterine rupture than in controls. Similar results were described by Sheiner et al. [13] who compared CTG tracings from 50 women with uterine rupture to 601 tracings from women with no uterine rupture. They observed that during the first stage of labour, abnormalities like tachycardia and reduced baseline variability were more frequently present in women with uterine rupture. However, only uterine tachysystole and disappearance of contractions occurred significantly more frequently in cases than in controls.

In our study, the presence of uterine tachysystole with or without relation to augmentation by oxytocin and/or induction of labour was not a predictor for uterine rupture. Pryor et al. [16] found a greater positive likelihood ratio (LR) for hyperstimulation within 2 hours prior to a uterine rupture in women undergoing TOLAC compared with women with a successful VBAC. Phelan and Settles [17] on the other hand did not find an association between abnormal uterine activity pattern and uterine rupture when comparing women with a uterine rupture to women with a spontaneous vaginal delivery or a successful VBAC. In our study, a proper assessment could not be made regarding the pattern of uterine activity in 28% of the tracings recorded during the last hour prior to uterine rupture. This could indicate that the circumstances that led to poor registration of uterine contractions in women with TOLAC were additional risk factors.

The strengths of this study are that it is population based, with a case and control group that are almost identical with regard to maternal demographics. Furthermore, because a strict definition was used, only complete uterine ruptures were included. Moreover, the assessors of the CTG tracings were completely blinded to the outcome by not only blinding clinical information but also the last part of the tracing that contained information on outcome because such information is known to cause bias in assessing CTG traces[18].

There are also some limitations to this study. At the time when the study was conducted, electronic storage of CTG was not implemented in the Danish labour wards. Thus, although most women attempting a TOLAC were monitored continuously during labour according to the guidelines, many of the recordings were lost or of poor quality. Thus, only a relative small amount of CTG recordings could be located. Furthermore, the quality of the tracings varied, and 13 CTG tracings lacked more than 30 minutes prior to the reported birth time. Likewise, the durations of CTG tracings available for evaluation varied and were in some cases less than 20 minutes. In general, our results might have yielded more significant values if the control group had been larger. The electronic storage of fetal heart rate monitoring that is performed today will hopefully lead to an easier and better assessment for comparison in future studies.

Our results confirm that the occurrence of pathological CTG tracings and severe variable decelerations may be predictors for uterine rupture. However, as the conditions are also present in a majority of controls, these findings could lead to a substantial increase in the rate of CD in women undergoing TOLAC, if used as the primary indicator of a uterine rupture. CTG interpretation is known to rely heavily on human factors which can have a profound effect on outcomes[19]. However, continuous surveillance with CTG in women with TOLAC is still the standard of care. The challenge of fetal signs of intolerance of labour occurring as frequently in cases as in controls should be addressed by continuous research in the field of electronic fetal monitoring to develop improved diagnostic possibilities as well as specific management algorithms for clinical situations with major diagnostic uncertainty.

In conclusion, a pathological CTG tracing should lead to awareness, but cannot provide the sole evidence of threatening uterine rupture and other risk factors or signs should be carefully evaluated.

## Supporting Information

S1 Appendix Classification of cardiotocographic tracings. S1 Appendix illustrates the evaluation scheme developed based on the International Federation of Gynaecology and Obstetrics (FIGO) guidelines.
(DOC)

S2 Appendix FIGO criteria for assessment of a cardiotocograph. S2 Appendix illustrates criteria’s for assessment of a cardiotocograph.
(DOC)

S1 Dataset Copy of the dataset underlying the study. (SAV)
