# Supplementary material for: Can Intrapartum Cardiotocography Predict Uterine Rupture among Women with Prior Caesarean Delivery?: A Population Based Case-Control Study
Source: PLoS One. 2016 Feb 12;11(2):e0146347. doi: 10.1371/journal.pone.0146347 (PMC4752316; doi:10.1371/journal.pone.0146347)
Supplement: S1 Appendix — illustrates the evaluation scheme developed based on the International Federation of Gynaecology and Obstetrics (FIGO) guidelines. (DOC) [file pone.0146347.s001.doc]

**S1 Appendix**

**Classification of cardiotocographic tracings:**

**Contractions:** (numbers) ______ per 10 minutes.

Sign of uterine hyperstimulation?: No ⬜ Yes ⬜

**Baseline fetal heart rate:** ______ bpm.

Episode of bradycardia: No ⬜ Yes ⬜ If Yes: (duration) ______ minutes.

**Variability:** Normal ⬜ Impaired ⬜ Increased ⬜

**Accelerations:** No ⬜ Yes ⬜ Numbers: ______

**Decelerations:** No ⬜ Yes ⬜ Numbers: ______

Uniform (write number): Variable (write number):

Early ⬜ Late ⬜ Mild ⬜ Severe ⬜

**Overall assessment of the cardiotocogram:**

Normal ⬜

Suspicious ⬜

Pathological ⬜

Preterminal ⬜
