# Supplementary material for: Can Intrapartum Cardiotocography Predict Uterine Rupture among Women with Prior Caesarean Delivery?: A Population Based Case-Control Study
Source: PLoS One. 2016 Feb 12;11(2):e0146347. doi: 10.1371/journal.pone.0146347 (PMC4752316; doi:10.1371/journal.pone.0146347)
Supplement: S2 Appendix — illustrates criteria’s for assessment of a cardiotocograph. (DOC) [file pone.0146347.s002.doc]

**S2 Appendix**

FIGO criteria for assessment of a cardiotocograph:

| **Parameter** | **Normal** | **Suspicious** | **Pathological** | **Pre-terminal** |
| --- | --- | --- | --- | --- |
| **Baseline Fetal Heart Rate** | 110-150 bpm^*^ | 100-110 or 150-170 bpm | < 100 or > 170 bpm | Complete absence of variability with or without decelerations or bradycardia. |
| **Variability (amplitude)** | 5-25 bpm | < 5 bpm or > 25 bpm for >40 min. | < 5 bpm for >40 min.  Sinusoidal pattern^**^ |
| **Decelerations** | None  or  Mild variable:  >15 bpm below baseline for >15 sec. | Sporadic decelerations of any type unless severe. | • Severe variable > 60 sec.  • Late decelerations  • Prolonged deceleration >3 minutes |
| **Accelerations** | ≥ 2 accelerations of ≥ 15 bpm for ≥ 15 sec. in 10 minutes. | < 2 accelerations of ≤ 15 bpm ≥ 15 sec. for >40 minutes. |  |

^*^ bpm = beats per minute.

^**^ A sinusoidal pattern is regular with cyclic changes in the fetal heart rate baseline, such as the sine wave. The characteristics of the pattern being: the frequency is less than 6 cycles/min, the amplitude is at least 10 beats/min and the duration should be 20 min or longer.

**Definition of pathological uterine activity**: more than five contractions per 10 minutes.
